# Oscillatory-Flow PCR Microfluidic Chip Driven by Low Speed Biaxial Centrifugation

**DOI:** 10.3390/bios13050555

**Published:** 2023-05-18

**Authors:** Yunlong Fan, Rongji Dai, Shuyu Lu, Xinyu Liu, Taiyan Zhou, Chunhua Yang, Xiaoming Hu, Xuefei Lv, Xiaoqiong Li

**Affiliations:** 1 Key Laboratory of Convergence Medical Engineering System and Healthcare Technology, the Ministry of Industry and Information Technology, 5 South Zhongguancun Street, Haidian District, Beijing 100081, China; 2 Beijing Key Laboratory for Separation and Analysis in Biomedicine and Pharmaceuticals, School of Life Sciences, Beijing Institute of Technology, 5 South Zhongguancun Street, Haidian District, Beijing 100081, China

**Keywords:** oscillatory-flow PCR, biaxial centrifugation, lab-on-a-disk, low centrifugal speed, microfluidic chip, microgravity, bubble

## Abstract

PCR is indispensable in basic science and biotechnology for in-orbit life science research. However, manpower and resources are limited in space. To address the constraints of in-orbit PCR, we proposed an oscillatory-flow PCR technique based on biaxial centrifugation. Oscillatory-flow PCR remarkably reduces the power requirements of the PCR process and has a relatively high ramp rate. A microfluidic chip that could perform dispensing, volume correction, and oscillatory-flow PCR of four samples simultaneously using biaxial centrifugation was designed. An automatic biaxial centrifugation device was designed and assembled to validate the biaxial centrifugation oscillatory-flow PCR. Simulation analysis and experimental tests indicated that the device could perform fully automated PCR amplification of four samples in one hour, with a ramp rate of 4.4 °C/s and average power consumption of less than 30 W. The PCR results were consistent with those obtained using conventional PCR equipment. Air bubbles generated during amplification were removed by oscillation. The chip and device realized a low-power, miniaturized, and fast PCR method under microgravity conditions, indicating good space application prospects and potential for higher throughput and extension to qPCR.

## 1. Introduction

Space microgravity and low radiation levels provide unique conditions for life science research [1,2]. PCR is indispensable in basic science and biotechnology for analyzing the expression of a moderate number of genes [3] in biological samples exposed to the space environment [4], environmental microbial communities in wastewater [5,6], and peripheral blood mononuclear cells of astronauts [7].

Space samples can be transported to the ground for PCR testing or tested in orbit using PCR devices on the International Space Station. For ground testing, months of in-orbit cryogenic storage are required from in-orbit sampling to ground analysis [8,9], raising concerns about the timeliness of data availability and quality of sample preservation [10]. Therefore, the results of in-orbit testing are immediately available and more accurate. However, manpower and resources are limited in space [1]. Limited manpower requires amplifying more samples with fewer operations to reduce the workload of the astronauts. Conventional PCR instruments operate through the cyclic heating and cooling of good thermal conductors and consume large amounts of power [11]. However, the power consumption and heat dissipation of space-station instruments are limited. Limited resources restrict the application of conventional PCR instruments, necessitating lower power to perform PCR and a faster ramp rate. The SmartCycler^®^ (Sunnyvale, CA, USA) and RAZOR^®^ EX (Salt Lake City, UT, USA) [3] take advantage of microfluidic chips, making it easier for small microreactors to obtain rapid ramp rates. However, bubbles produced during amplification interfere with the accuracy of the results to some extent and decrease the success rate of in-orbit PCR tests [10,12]. Therefore, PCR technology with portable features, such as small size and low power consumption, is the key to solving the problem of in-orbit detection.

In oscillatory flow PCR, the sample is moved between different desired temperature zones to complete a temperature cycle [13,14,15]. Oscillatory-flow PCR has two main implementation methods: fixed temperature zones with liquid movement [15,16,17,18,19] and fixed liquids with temperature zone movement [13,14]. Fixing the liquid can reduce the complexity of the microfluidic design; however, it cannot increase the heat conduction rate effectively. Fixing temperature zones prevents the bottom of the chip from participating in the process of thermal transition so that the liquid can be heated or cooled quickly [20].

In this study, with the aim of solving the in-orbit PCR problems considering manpower and resource constraints, a new method was developed and evaluated. The principle is oscillatory-flow PCR with fixed temperature zones and liquid oscillation, driven by centrifugal forces in two different directions on a microfluidic chip. To verify the biaxial centrifugation oscillatory-flow PCR, a microfluidic chip that could perform dispensing, volume correction, and oscillatory-flow PCR of four samples simultaneously and an automatic biaxial centrifugation device were designed and assembled. Through simulations and testing, low-consumption centrifugation and temperature control were achieved. Comparative experiments were performed using conventional PCR instruments, and bubble removal was observed.

## 2. Materials and Methods

### 2.1. Biaxial Centrifugation Analysis

#### 2.1.1. Chip Fabrication

**Figure 1:**
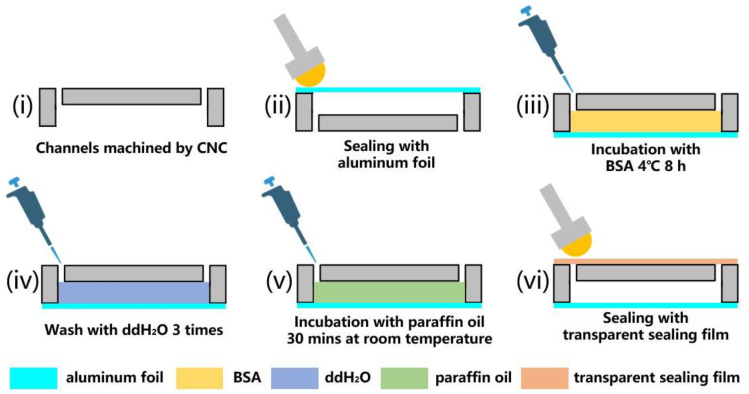
Flowchart of fabrication of chip: (**i**) channels machined using CNC; (**ii**) sealing with aluminum foil; (**iii**) incubation with BSA 4 °C 8 h; (**iv**) wash with ddH_2_O; (**v**) incubation with paraffin oil for 30 min at room temperature (20 °C–25 °C); (**vi**) sealing with transparent sealing film.

**Figure 2:**
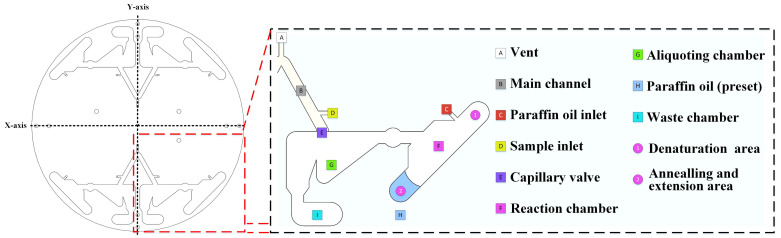
Diagram of the different parts (areas) of the chip and their function.

The chip was designed using the SolidWorks software (2016 SP04) for three-dimensional structural drawing. As the channels were single-layer structures, they were etched using CNC and drilled. Considering the heat deflection temperature and light transmittance requirements, polycarbonate was chosen as the chip material. An aluminum foil sealing film (YA0240-S, Solarbio, Beijing, China) was used for sealing the side of the etched channel, and a roller was used for flattening and tightening. The surface treatments included BSA coating and paraffin oil lubrication. BSA (A8020; Solarbio, Beijing, China), at a concentration of 1 mg/mL, was added to fill the chip. Then, the chip was incubated at 4 °C for 8 h. After incubation, the chips were drained by shaking. The chip channel was then washed thrice using ddH_2_O. After emptying, the reaction chamber was filled with paraffin oil (B500301, Sangon, Shanghai, China) through the paraffin oil inlet and incubated for 30 min. A pipette was used to drain as much paraffin oil as possible through the channels. The small amount of residue did not affect the amplification process. Small pieces of a transparent sealing film were used to seal the paraffin oil inlet. The four regions of the chip were handled similarly. A flowchart of the chip fabrication process is shown in Figure 1. The diagram of the different parts (areas) of the chip is shown in Figure 2.

#### 2.1.2. Centrifugal Drive and Measurement

A simple platform was constructed to observe the time required for the liquid to move during centrifugation. The platform consisted of a DC motor (GA42Y, Xinyongtai, Shenzhen, China), motor driver, high-speed camera (Al-030U780M, Hikvision, Hangzhou, China), and a supporting structure. The motor driver has a built-in regulation algorithm that can drive the DC motor to achieve a fixed centrifugal speed. The actual speed was measured using a handheld tachometer (DLY-2301; DELIXI, Hangzhou, China). The high-speed camera had a frame rate of 100 fps and was used to capture the liquid movement after starting the centrifugation. The time interval between the frames was 10 ms. The liquid movement time was calculated as the difference between the first frame and the end of the frame liquid movement. The centrifugal speed of the device mentioned in Section 3.2 was also measured using a handheld tachometer. The power consumption of the centrifugation device was recorded using a power meter (Weitai, Quzhou, China). When the centrifugation was stable, the number shown on the power meter was recorded three times, and the average was calculated.

### 2.2. Device Verification with Chip

#### 2.2.1. Temperature Distribution and Ramp Rate Measurement

For the temperature distribution measurement, the actual temperature was measured using a thermal imaging camera (Ti25, FLUKE, Everett, WA, USA) to capture the temperature at the end of the copper columns, and a thermal imager supporting software (SmartView, FLUKE, Everett, WA, USA) was used for the numerical analysis of the temperature point.

A handheld thermometer (FLUKE 51-II, FLUKE, Everett, WA, USA) equipped with a small J-type thermocouple probe (80PJ-J, FLUKE, Everett, WA, USA) was used for liquid temperature measurements. After the chip was fabricated, holes were drilled above denaturation areas and annealing and extension areas. After fixing the chip on the heating module, 20 μL of water of the initial temperature was pipetted into the hole. The thermocouple probe was immediately inserted into the hole to contact the water, and the time was recorded using a stopwatch to estimate the ramp rate.

#### 2.2.2. Temperature Control Stability and Power Consumption Measurement

A PID algorithm was used for constant temperature control. The temperature data and duty cycles were transmitted back to the host computer through a serial port at a frequency of 2 Hz and recorded. Continuously, 1500 s of data were recorded and used to calculate the temperature stability and power consumption. The stability was evaluated by the fluctuation of the temperature curve between the maximum and minimum values of the curve. Power consumption was evaluated using the average duty cycle of the PID process. The power consumption was calculated using Equation (1).

(1) $$\bar{P}=P_{\max}\times \bar{\eta }$$

where $\bar{P}$ is the power consumption, *P*_max_ is the maximum power of the heating film, and $\bar{\eta }$ is the average cycle when the temperature stabilizes.

#### 2.2.3. Template and Amplification Reagent

Three genes of typical microbes found in the space station environment were chosen as targets: arcC from Staphylococcus aureus (240 bp), yhaI from Klebsiella pneumonia (249 bp), and PEX-2 from Penicillium (177 bp). Plasmids containing the corresponding sequences were used for testing. All plasmids and primers were designed manually and synthesized by Biotech. The primer sequences used were as follows:

arcC-forward: 5′-TGGTGCAATGTCACAGGGTA-3′;
arcC-reverse: 5′-CCACGTCCTGCATCTTCTTT-3′;
yhaI-forward: 5′-ATTTGAGCGGCTGGAAAGAG-3′;
yhaI-reverse: 5′-AGCGGCCGATATCATGCAT-3′;
PEX-2-forward: 5′-CGATCAGTCCCCAAGTGAAT-3′;
PEX-2-reverse: 5′-AGTCTACCGGTGCAACCAAC-3′.

The Talent Quantitative Assay Kit (FP209, Tiangen, Beijing, China) was used to prepare 20 μL PCR reactions for all PCR tests. The reaction contained 7.8 μL Talent qPCR PreMix (SYBR GREEN), 10 μL RNase-Free ddH_2_O, 0.6 μL forward primer, 0.6 μL reverse primer, and 1 μL template.

#### 2.2.4. PCR Process and Agarose Gel Electrophoresis Test

The PCR experiments were performed simultaneously on the chip using a PCR instrument (Gentier 96 real-time PCR system, Tianlong, Xi’an, China). The PCR conditions were as follows: 40 cycles of initial denaturation at 94 °C for 5 min, denaturation at 94 °C for 30 s, and annealing and extension at 54 °C for 45 s. The PCR reagents were pipetted into the main channel of the chip. Then, the chip was placed on the device, and the process proceeded automatically. The theoretical size of amplicons was 127 bp. The results were tested using agarose gel electrophoresis (2% w/v) as the standard method to analyze the size of the DNA amplicons. A 100 bp DNA Ladder (MD109-01, Tiangen, Beijing, China) was used as a marker for product size determination.

## 3. Results and Discussion

### 3.1. Chip Design and Centrifugal Drive Validation

#### 3.1.1. Chip Design and Workflow

The whole process was performed on the chip after sample injection, including dispensing, volume correction, and oscillatory flow amplification. The overall design is illustrated in Figure 2. Vent (A) is a hole that provides air when the liquid in the main channel moves through the capillary valve. The main channel (B) held the liquid when the PCR reaction sample was added through the sample inlet. The paraffin oil inlet (C) is a hole for paraffin oil injection. The sample inlet (D) is a hole for the PCR reaction sample injection. A capillary valve (E) was used to block the PCR reaction sample during injection. A reaction chamber (F) was used for oscillatory-flow PCR. It has a denaturation area ① and an annealing and extension area ②. The temperature of the denaturation area was approximately 94 °C, and the temperature of the annealing and extension area was approximately 54 °C. The angle of the reaction chamber to the centrifugal axis was 45°. An aliquot chamber (G) was used to adjust the volume to 20 μL and was linked to the main channel with a capillary valve to avoid uneven liquid separation [21,22]. Paraffin oil (H) was used to lubricate and avoid evaporation of the PCR reaction sample during the PCR. Waste chamber (i) held the excess PCR reaction sample during dispensing.

**Figure 3:**
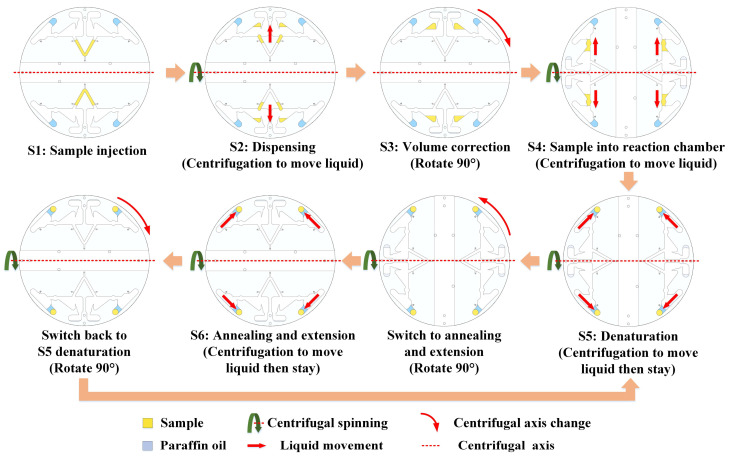
Workflow of the chip.

When the liquid was pipetted into the main channel and the chip was placed in the device, all processes were automatically completed by the device. The centrifugal axis is a straight line passing through the center of the chip (red dashed line in Figure 3). The device changed the direction of the centrifugal force on the liquid by changing the centrifugal axis. A stepper motor was used to rotate the chip and change its centrifugal axis.

S1: Sample injection: The injected sample was a PCR reaction sample (containing templates and primers), and the movement of the liquid was blocked using a capillary valve after injection and remained in the main channel. When filling the channel through the sample inlet, air was exhausted from the vent of the channel on the other side. Paraffin oil was also added to the reaction chamber through the paraffin oil inlet.

S2: Dispensing: This step represents the initial PCR reaction sample distribution. When the chip is centrifuged, the PCR reaction sample travels through the capillary valve into an aliquoting chamber. Simultaneously, the paraffin oil moves to the annealing and extension sides of the reaction chamber.

S3: Volume correction: The volume of the aliquoting chamber is only 20 μL, and the excess will drop into the waste chamber. After volume correction, the stepper motor rotates the chip 90° to change the centrifugal axis.

S4: Sample into reaction chamber: After the centrifugal axis was changed, the chip was centrifuged, and the PCR reaction sample in the aliquoting chamber and paraffin oil moved to the denaturation side of the reaction chamber.

S5: Denaturation: This step follows steps S4 or S6. After S4, the PCR reaction sample containing paraffin oil was in the denaturation area. The samples were heated to the required temperature for denaturation and maintained for a certain time. After S6, additional centrifugation was performed to move the PCR reaction sample containing paraffin oil to the denaturation area. After denaturation, the stepper motor rotated the chip 90° to change the centrifugal axis.

S6: Annealing and extension: After the centrifugal axis was changed, the chip was centrifuged, and the PCR reaction sample with paraffin oil was moved to the annealing and extension areas and cooled to the temperature required for annealing and extension for a certain time. After annealing and extension, the stepper motor rotated the chip 90° to change the centrifugal axis changed and then returned to S5 as a cycle.

This chip design represents an initial exploration of space PCR applications. Considering the inconvenience of operating in a space environment, it is preferable to perform the experimental operations in fewer steps. Therefore, the dispensing and volume correction functions were designed to serve as the basis for future extensions. In subsequent studies, we will develop a method for amplification reactions using a single pipette with primer preset on the chip.

#### 3.1.2. Centrifugal Drive Validation

**Figure 4:**
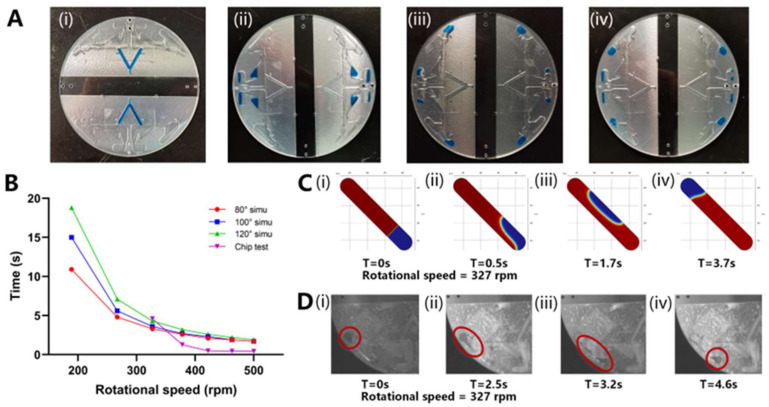
(**A**) Photo of the liquid movement flow in the chip: (i) When the sample was injected into the chip; (ii) After dispensing; (iii) After the sample entered the reaction chamber/Denaturation (iv) Annealing and extension. (**B**) The relationship between liquid movement time and centrifugal speed under simulation and test conditions. (**C**) Simulation of liquid movement process at 327 rpm centrifugal speed: (i) Liquid state at 0 s; (ii) Liquid state at 0.5 s; (iii) Liquid state at 1.7 s; (iv) Liquid state at 3.7 s. (**D**) Photos of liquid movement process at 327 rpm centrifugal speed: (i) Liquid state at 0 s; (ii) Liquid state at 2.5 s; (iii) Liquid state at 3.2 s; (iv) Liquid state at 4.6 s.

There are four processes in which the liquid inside the chip is driven by the centrifugal force: (a) from the sample inlet channel to the aliquoting chamber, (b) from the aliquot chamber to the reaction chamber, (c) from the denaturation area to the annealing and extension area, and (d) from the annealing and extension area to the denaturation area. Referring to the design principle of capillary valves [23,24], the vent was designed close to the x-axis, and the capillary valve was designed far from the x-axis so that a greater pressure on the liquid could be reached at a lower centrifugal speed. Figure 4A shows the process of the liquid moving from the sample injection chamber to the sample reaction chamber and the oscillating steps. Figure 4A(i) shows the initial state when the sample was injected into the chip (S1). Figure 4A(ii) shows the state after dispensing (S2) and volume correction (S3). Figure 4A(iii) shows the state after the sample entered the reaction chamber (S4) and was ready for denaturation (S5). Figure 4A(iv) shows the state during annealing and extension (S6). It was verified that 500 rpm drove the liquid through the capillary valve between S1 and S2. All liquid movement steps were performed at 500 rpm.

The time of liquid movement from one site to the other in the reaction chamber influenced the average ramp rate and the total time of the PCR experiment. In addition, a higher centrifugal speed requires better stability and greater electrical power consumption. Therefore, faster liquid movement at a relatively low speed is the goal of the optimization.

Through a simulation, we preliminarily confirmed the relationship between the centrifugal speed, the contact angle of the chip material, and liquid movement time, as shown in Figure 4A. The simulated liquid movement is shown in Figure 4B,C. When the centrifugal speed was lower than 327 rpm, the contact angle of the material influenced the speed of the liquid movement. When the centrifugal speed was higher than 327 rpm, the difference in the time of liquid movement was within 1 s. As the centrifugal speed increased, the liquid movement time continuously decreased, and when the centrifugal speed was higher than 422 rpm, the time for liquid movement was less than 2 s.

Subsequently, the actual liquid movement process in the chip and time were recorded, as shown in Figure 4D. When the centrifugal speed was <327 rpm, the liquid did not move. The inability to move at low speed might be because the chip was processed using the CNC method, and the roughness of the inner wall of the chip formed a certain static resistance to the liquid movement. When the centrifugal speed was higher than 422 rpm, liquid movement occurred within 1 s.

The simulation and measured results show that when the speed was over 327 rpm, the liquid moved at a faster speed, and the difference between the contact angles was no longer evident. Therefore, by positioning the centrifugal speed at the minimum value of the interval in which the centrifugal force played a leading role, the power consumption and liquid movement speed were optimized.

### 3.2. Device Design

#### 3.2.1. Biaxial Centrifugation Module

**Figure 5:**
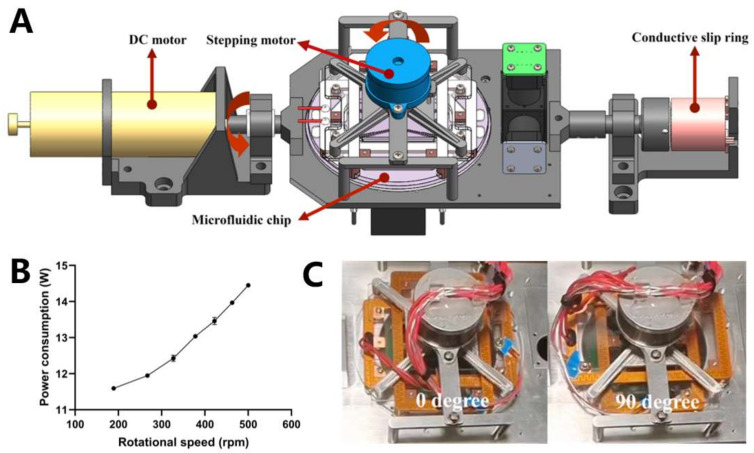
(**A**) Three-dimensional diagram of the structure of the biaxial centrifugal mechanism. (**B**) The relationship between centrifugal speed and power consumption of the centrifugal system. (**C**) Photos of the structure at rotation 0° and 90°.

Biaxial centrifugation uses a DC motor to provide a high speed and a stepper motor for angle switching. The heating module, control circuit, and stepper motor were fixed to an aluminum alloy plate. The chip was then placed on a heating module connected to the stepper motor rotor. The stator of the stepper motor was fixed using an aluminum alloy plate. The centrifugal axis of the DC motor coincided with the centrifugal axis of the chip. When the DC motor centrifuges the aluminum alloy plate, the liquid in the chip is subjected to an axial, centrifugal force. When the stepper motor rotated the heating module to switch angles, the chip was rotated 90° clockwise or counterclockwise to complete the centrifugal axis switching. A counterweight structure was used to balance the structural center of gravity. On the opposite side of the DC motor, a slip ring was used to power the electrical appliances on the centrifugal structure and provide control commands. A three-dimensional diagram of the biaxial centrifugal mechanism is shown in Figure 5A.

After completing assembly, the device was tested for operation at 189–500 rpm. The tests were conducted at 0° and 90°, as shown in Figure 5C. The device operated stably at the test speeds. The motor power at different speeds was measured using a power meter, as shown in Figure 5B. The motor power gradually increased with increasing speed. Considering the liquid movement results described in Figure 4B, the device used a centrifugal speed of 422 rpm for the two axial centrifuges.

For many lab-on-a-disk devices, the transfer of liquid between chambers requires thousands of revolutions per minute [25,26]. For the biaxial centrifugation oscillatory-flow PCR device, the centrifugal force was higher owing to the presence of stepper motors and temperature control systems, which may lead to higher power consumption and a larger size of the structure for stabilization. To meet the requirements of space applications for low power consumption and small size, the centrifugation speed of all the liquid movement processes in the chip was designed to be approximately 500 rpm.

#### 3.2.2. Temperature Control Module

Oscillatory-flow PCR requires the device to provide two constant-temperature zones between which the liquid oscillates to meet the requirements for liquid temperature change. Therefore, the design of the two constant-temperature zones must meet two requirements: (1) high-stability temperature regions and (2) no mutual influence between the temperature regions. Because the thermal structure was in close contact with the chip and needed to be centrifuged, the size and weight needed to be as small as possible to reduce the centrifugal load on the device.

**Figure 6:**
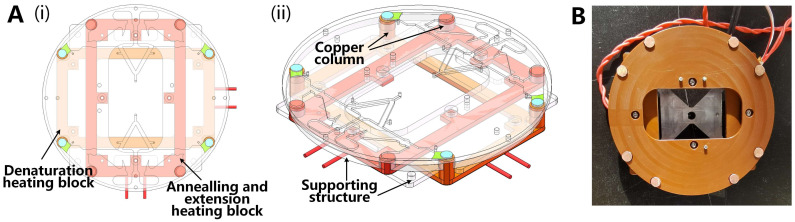
(**A**) Above and side view of thermal structure with chip on it: (i) Structural view; (ii) Structure 45° side view. (**B**) Photo of above view of thermal structure.

Two independent copper blocks were used to provide constant-temperature zones for denaturation and annealing. Each copper block was rectangular with four columns. The two copper blocks were fixed on the polyimide structures, and the tops of the columns were placed on the same plane to hold the chip. Heating films were used as the heat source and placed at the bottom of the copper block using a PT1000 temperature sensor. The circuit system collects temperature data and controls the output power of the heating film using a PID feedback algorithm for temperature control. The structural design is shown in Figure 6A. A photograph of the thermal structure is presented in Figure 6B.

#### 3.2.3. Circuit Module and Device Assembly

**Figure 7:**
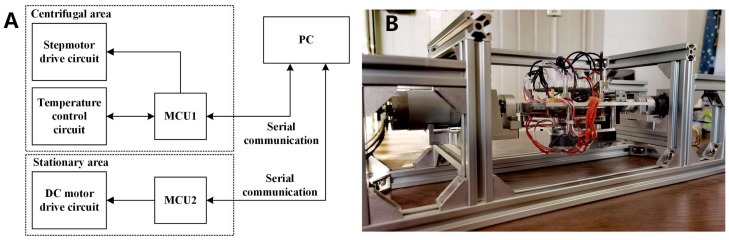
(**A**) Block diagram of the electronic control system. (**B**) The structure of the whole instrument.

The device is divided into centrifugal and stationary areas. In the centrifugal area, MCU1 was used to control the stepper motor and temperature. MCU2 was used to control the DC motor in the stationary area. The two MCUs were connected to a PC through serial communication. MCU1 uploads the temperature data, duty cycle data, and current experimental cycle status. MCU2 receives PC commands and controls the DC motor centrifuging at a specified speed for a certain time. A block diagram of the electronic control system is presented in Figure 7A. The instrument structure is shown in Figure 7B. The size of the device was 450 mm × 165 mm × 165 mm, and it weighed less than 5 kg.

### 3.3. Device Verification with Chip

#### 3.3.1. Temperature Distribution and Ramp Rate Measurement

**Figure 8:**
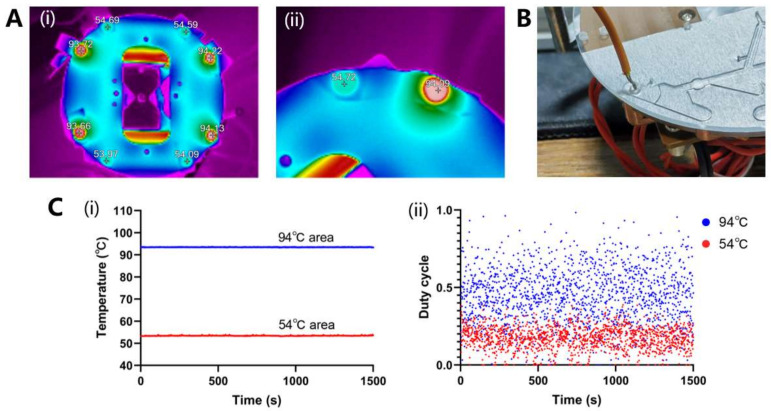
(**A**) The temperature distribution was captured with a thermal imaging camera and displayed using software; (i) Temperature distribution over the entire thermal structure; (ii) Temperature distribution of a set of 94 °C and 54 °C. (**B**) Photo of ramp rate measurement in the chip. (**C**) (i) The constant temperature curve of two areas. (ii) The duty cycle curve of two areas.

The temperature distribution was captured using a thermal imaging camera and displayed using software, as shown in Figure 8A. The temperature accuracy and uniformity at the end of the copper column were within ±1.0 °C.

For ramp rate measurement, the 54 °C water was pipetted into the 94 °C area and temperature change was measured using a handheld thermometer, as shown in Figure 8B. It took about 8 s for the water to heat to 94 °C. The water of 94 °C was pipetted in the 54 °C area, and within approximately 8 s, it cooled down to 54 °C. Finally, according to Equation (S1), the up ramp and down ramp of the oscillatory-flow PCR device was 4.4 °C/s.

#### 3.3.2. Temperature Control Stability and Power Consumption Measurement

The constant-temperature curve over 1500 s determined using the temperature sensor is shown in Figure 8C(i). The temperature fluctuation of the two zones was all within ±0.1 °C, which means temperature control had good stability. Finally, the power consumption was evaluated using a duty cycle of more than 1500 s, as shown in Figure 8C(ii). The rated power of the heating film used in the 94 °C temperature zone was 24 W, and the average duty cycle was 44.7%. The rated power of the heating film used in the 54 °C temperature zone was 12 W, and the average duty cycle was 17.2%. The total average power was calculated as 12.8 W using Equation (1).

#### 3.3.3. Verification of Oscillatory-Flow PCR

**Figure 9:**
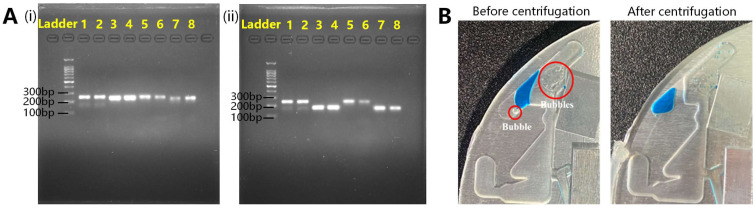
(**A**) The gel electrophoresis results of PCR verification. (i) Lanes 1 and 2 are the yhaI results on conventional PCR device; lanes 3 and 4 are the arcC results on conventional PCR device; lanes 5 and 6 are yhaI results on chip; lanes 7 and 8 are arcC results on chip. (ii) Lanes 1 and 2 are yhaI results on a conventional PCR device; lanes 3 and 4 are PEX-2 results on a conventional PCR device; lanes 5 and 6 are yhaI results on a chip; lanes 7 and 8 are PEX-2 results on a chip. (**B**) Photo of bubble removal before and after centrifugation.

After the device was built, as described in Section 3.2, a PCR experiment on the chip was used to verify the device. The experimental procedure is described in Section 2.2.4. and shown in Video S1. PCR on the chip and on a conventional PCR device were performed simultaneously. The gel electrophoresis results are shown in Figure 9A. In Figure 9A(i), lanes 1 and 2 are the yhaI results on the conventional PCR device; lanes 3 and 4 are the arcC results on the conventional PCR device; lanes 5 and 6 are the yhaI results on the chip; lanes 7 and 8 are the arcC results on the chip. In Figure 9A(ii), lanes 1 and 2 are yhaI results on the conventional PCR device; lanes 3 and 4 are PEX-2 results on the conventional PCR device; lanes 5 and 6 are yhaI results on the chip; lanes 7 and 8 are PEX-2 results on the chip. The amplification results showed that repeated experiments (yhaI), as well as different amplification targets, could be amplified using this system, indicating good PCR performance.

The entire experimental process required less than 60 min. Compared to conventional PCR instruments, shorter time consumption benefits from the liquid’s direct contact with the heat source, providing a faster rate of temperature change. According to the power consumption test described in Section 3.2.1 and Section 3.3.2, the average power consumption was only approximately 30 W. Compared to miniPCR (miniPCR bio, Cambridge, MA, USA), biaxial centrifugation oscillatory-flow PCR has the advantages of reduced power consumption and faster ramp rate. Owing to power consumption limitations, PCR devices that use conventional electric heating and cooling devices cannot achieve high ramp rates. An insufficient ramp rate leads to a longer amplification time, resulting in larger total power consumption. Biaxial centrifugation oscillatory-flow PCR offers a new method for achieving a high ramp rate with relatively low power consumption, which is suitable for space applications.

Air bubbles in the chamber were eliminated during biaxial centrifugation, as shown in Figure 9B. If the bubbles formed during the heating step could not be removed, they would accumulate and affect the heat transfer from the heat source to the liquid, resulting in PCR failure. For every step, the time before bubble generation was sufficient for PCR. Removing bubbles between steps in step was sufficient for successful PCR, which was verified.

## 4. Conclusions

In view of the problems faced by spatial nucleic acid amplification experiments, such as limited power consumption, volume, and microgravity environments, we designed a biaxial centrifugation oscillatory-flow PCR device based on microfluidic chips. This chip and device could not only quickly complete the PCR of four samples simultaneously under low power consumption but also reduce the operation process to a certain extent. Air bubbles generated during PCR were eliminated during the oscillating process.

By simulating the oscillation process of biaxial centrifugation driving liquids and testing with a high-speed camera, we determined that low-speed centrifugation of only 422 rpm could drive liquids between temperature zones within 1 s. Through a simulation and an actual test of the liquid up-and-down ramps in the chip, the reason for the rapid temperature change was explained. The average ramp rate of the oscillating PCR process could reach 4.4 °C/s, allowing PCR experiments to be completed in less than 60 min. The size of the device was 450 mm × 165 mm × 165 mm, its weight was less than 5 kg, and its average power consumption was less than 30 W during operation. Comparison of the amplification experiments on the chip with the device with those on a PCR instrument using gel electrophoresis showed that the biaxial centrifugation oscillatory-flow PCR device results were consistent with those of conventional PCR instruments.

The chip and device represent a low-power, miniaturized, and fast PCR implementation method under microgravity conditions, which has good space application prospects and the potential to further improve throughput and expand to qPCR.

## Data Availability

The data presented in this study are available on request from the corresponding author.

## Supplementary Materials

The following supporting information can be downloaded at: https://www.mdpi.com/article/10.3390/bios13050555/s1, Video S1: Process of biaxial centrifugation. Figure S1. (A) Simulation module of liquid oscillation. (B) Simulation module of temperature change in reaction chamber. (C) Simulation module of temperature distribution on chip. Figure S2. (A) (i) Temperature distribution of liquid at 54 °C when it reaches the 94 °C temperature zone for 0.1 s. (ii) Illustration of measure points selection. (B) Comparison of up and down ramp between no compartment and 0.2 mm PC. Figure S3. (A) Simulation result of temperature distribution in the plane where the liquid in the chip was located. (B) The temperature points on the straight line shown by the yellow arrow in Figure S3A.

## Abbreviations

PCR: polymerase chain reaction; CNC: computerized numerical control machine; BSA: bovine serum albumin; DC: direct current; PID: proportional integral derivative; MCU: microcontroller unit; PC: personal computer.
