# Supplementary material for: Oscillatory-Flow PCR Microfluidic Chip Driven by Low Speed Biaxial Centrifugation
Source: Biosensors (Basel). 2023 May 18;13(5):555. doi: 10.3390/bios13050555 (PMC10216608; doi:10.3390/bios13050555)
Supplement: Supplementary file 1 [file biosensors-13-00555-s001.zip › biosensors-2345888-supplementary-for publish.pdf]

biosensors
MDPI

Supporting Information

Oscillatory-Flow PCR Microfluidic Chip Driven by Low Speed Biaxial Centrifugation

Yunlong Fan [1,2], Rongji Dai [1,2], Shuyu Lu [1,2], Xinyu Liu [1,2], Taiyan Zhou [1,2], Chunhua Yang [1,2], Xiaoming Hu [1,2], Xuefei Lv [1,2] and Xiaoqiong Li [1,2,*]

1 Key Laboratory of Convergence Medical Engineering System and Healthcare Technology, the Ministry of Industry and Information Technology, 5 South Zhongguancun Street, Haidian District, Beijing 100081, China
2 Beijing Key Laboratory for Separation and Analysis in Biomedicine and Pharmaceuticals, School of Life Sciences, Beijing Institute of Technology, 5 South Zhongguancun Street, Haidian District, Beijing 100081, China
* Correspondence: aeople@126.com

1. Liquid Oscillation Simulation

Simulation was used to explore the rate at which liquid moves over temperature zones. The simulation model used the two-phase flow model from the COMSOL multiphysics. The two phases were set to water and air. The water was set as laminar flow property and the force was constant. As shown in Figure.S1(A), the force direction of the liquid was set vertical to the rotational axis, and the angle between the channel and the rotational axis was 45°. The force of the four regions on chip were axisymmetric so that only one of them was simulated. The relationship between force on liquid *F* and rotational speed ω was shown as Equation.2. Rotational speed was set from 189 rpm to 500 rpm (equivalent to gravity 1g to 7g). The gas–liquid interface adopted the phase field configuration. The contact angle was set to 80°, 100°, and 120° to explore the influence of hydrophobic properties of chip materials on the liquid movement. After solving the phase field by phase initialization, the transient change was solved in steps of 0.1s, and the length of time from the liquid to the other end from the initial state was recorded.

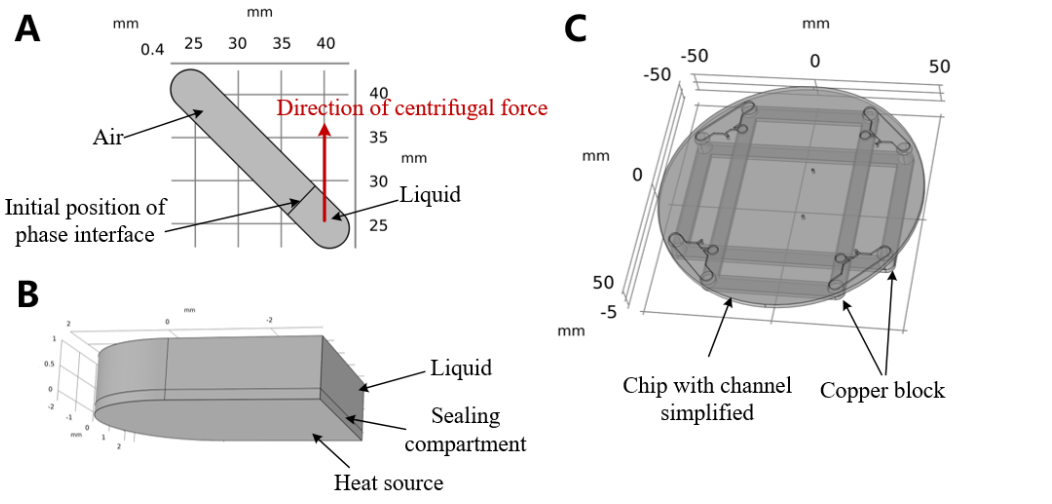

Figure S1. (**A**) Simulation module of liquid oscillation. (**B**) Simulation module of temperature change in reaction chamber. (**C**) Simulation module of temperature distribution on chip.

2. Temperature Change Simulation

Simulation was used to explore temperature change of liquid in different zones. The simulation model used was heat transfer in solids and fluids. To reduce the amount of

calculation, only liquid involved was simulated locally. The model is shown in Figure.S1(B). Two situations of heat transfer were simulated, heat transfer with sealing film and without sealing film. The sealing film material was set to polycarbonate whose thermal conductivity was 0.2 W/(m·K) and the thickness was 0.2mm. Since the material of chip was polycarbonate, the thermal conductivity of the rest of the liquid surface was also set to the PC thermal conductivity. The heat source was set as a constant temperature plane and the temperature was set to the target temperature. The liquid temperature and ambient temperature were initialized to the starting temperature. The simulation process included the process of raising the liquid from the starting temperature of 54°C to the target temperature of 94°C and cooling from the starting temperature of 94°C to the target temperature of 54°C. The simulation results were the temperature distribution of the liquid from 0 seconds to the 20th second, with a step interval of 1 second. Points in the liquid at different heights from the heat source were taken to calculate the average value to represent the temperature of the liquid.

The ramp rate is an important criterion for evaluating PCR systems. Faster ramp rates mean faster PCR experiments. In oscillatory-flow PCR, the average ramp rate v_T can be expressed as Equation.S1.

$$v_T = \frac{\Delta T}{t_1 + t_2}. \tag{S1}$$

ΔT represents the temperature difference, t_1 represents the time it takes for the liquid to move, and t_2 represents the time it takes for the liquid to change from previous temperature to next temperature. The time for liquids to move in the temperature zone was analyzed in Section 3.1.3. The time for the liquid to heat or cool was evaluated through simulation. When the 54°C liquid reached the 94°C temperature zone, the liquid temperature showed gradient conduction from the bottom up. Five temperature measurement points at different heights of the liquid were used to evaluate the temperature change of the liquid, as shown in Figure.S2(A). The results that Figure.S2(B) showed were comparisons of ramp rate between direct contact and 0.2mm polycarbonate. For oscillatory-flow PCR, the liquid was directly in contact with the area of constant temperature so that target temperature could be reached in 7 seconds. In contrast, with conventional temperature variation method, the ramp rate would be much lower even if only 0.2mm PC material was used to isolate the liquid and heat source. This is also the main reason why oscillatory-flow PCR can be fast. The process of cooling from 94 °C to 54 °C was symmetrical with the process of rising from 54 °C to 94 °C, and the results were basically the same.

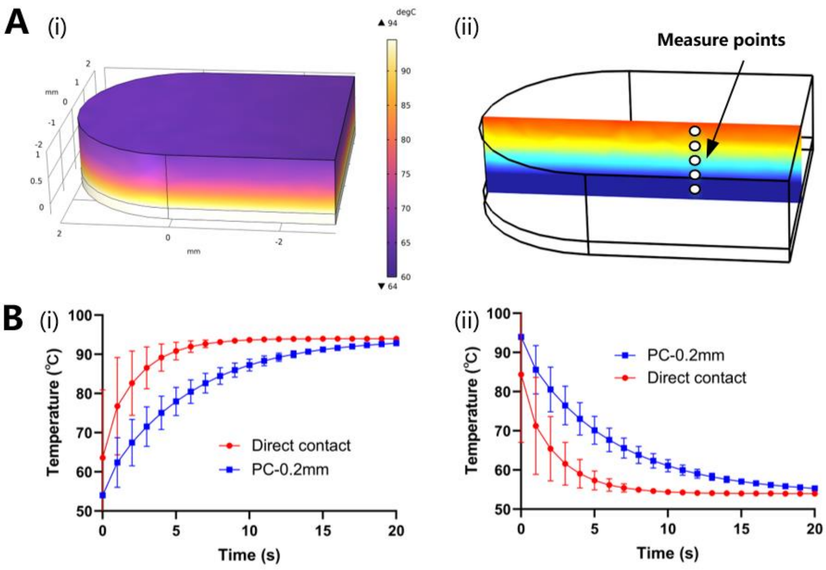

Figure S2. (**A**) (i) Temperature distribution of liquid at 54°C when it reaches the 94°C temperature zone for 0.1s. (ii) Illustration of measure points selection. (**B**) Comparison of up and down ramp between no compartment and 0.2mm PC.

3. Temperature Distribution Simulation

Simulation was used to explore temperature uniformity of different zones. The simulation model used was heat transfer in solids. The model is shown in Figure.S1(C). To reduce the amount of calculation, the chip was simplified so that only four reaction chambers were simulated. The model of the heating blocks was set the same as the structure, as shown in Figure.6(A)(i) and (ii). The two copper blocks were set to 94°C and 54°C constant temperature heat sources, and the ambient temperature was 20°C. The simulation results were the on-chip thermal distribution at steady state.

Simulation was used to evaluate the temperature consistency at the ends of each column with chip. Figure.S3(A) showed the temperature distribution in the plane where the liquid in the chip was located. As can be seen from the figure, the end of the copper column could provide a uniform temperature environment to the liquid inside the chip and the two temperature regions have no obvious mutual influence. Figure.S3(B) showed the temperature points on the straight line shown by the yellow arrow in Figure.S3(A). The temperature curve along the reaction chamber further proved that there is no mutual interference between the two temperature zones.

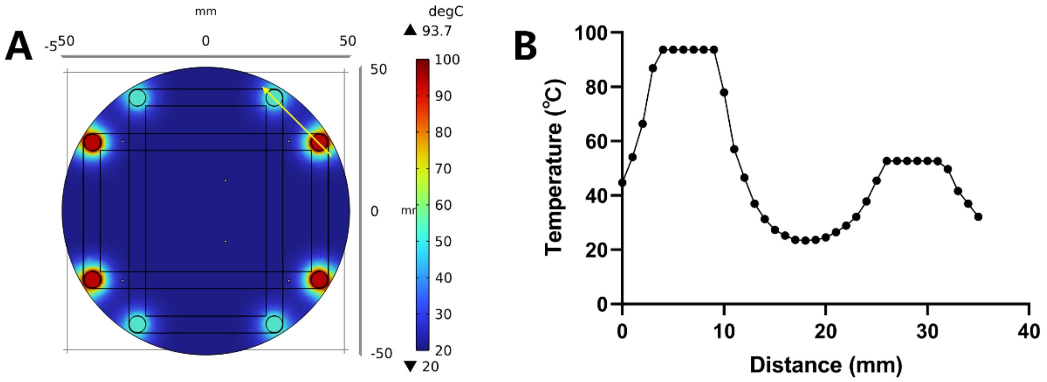

Figure S3. (**A**) Simulation result of temperature distribution in the plane where the liquid in the chip was located. (**B**) The temperature points on the straight line shown by the yellow arrow in Figure.S3(A).
